# The Relationship between Social Networking Site Use and the Internalization of a Thin Ideal in Females: A Meta-Analytic Review

**DOI:** 10.3389/fpsyg.2017.01351

**Published:** 2017-08-07

**Authors:** John Mingoia, Amanda D. Hutchinson, Carlene Wilson, David H. Gleaves

**Affiliations:** ^1^School of Psychology, Social Work and Social Policy, University of South Australia, Adelaide SA, Australia; ^2^Flinders Centre for Innovation in Cancer, Flinders University, Adelaide SA, Australia; ^3^Cancer Council South Australia, Adelaide SA, Australia

**Keywords:** social networking, eating disorders, internalization, Facebook, body image

## Abstract

Previous research has indicated that exposure to traditional media (i.e., television, film, and print) predicts the likelihood of internalization of a thin ideal; however, the relationship between exposure to internet-based social media on internalization of this ideal remains less understood. Social media differ from traditional forms of media by allowing users to create and upload their own content that is then subject to feedback from other users. This meta-analysis examined the association linking the use of social networking sites (SNSs) and the internalization of a thin ideal in females. Systematic searches were performed in the databases: PsychINFO, PubMed, Web of Science, Communication and Mass Media Complete, and ProQuest Dissertations and Theses Global. Six studies were included in the meta-analysis that yielded 10 independent effect sizes and a total of 1,829 female participants ranging in age from 10 to 46 years. We found a positive association between extent of use of SNSs and extent of internalization of a thin ideal with a small to moderate effect size (*r* = 0.18). The positive effect indicated that more use of SNSs was associated with significantly higher internalization of a thin ideal. A comparison was also made between study outcomes measuring broad use of SNSs and outcomes measuring SNS use solely as a function of specific appearance-related features (e.g., posting or viewing photographs). The use of appearance-related features had a stronger relationship with the internalization of a thin ideal than broad use of SNSs. The finding suggests that the ability to interact with appearance-related features online and be an active participant in media creation is associated with body image disturbance. Future research should aim to explore the way SNS users interact with the media posted online and the relationship linking the use of specific appearance features and body image disturbance.

## Introduction

Media consumption is rising worldwide, with the average person engaging in over 10 hours of media use per day (Nielsen, 2016). Extended exposure to media can have negative implications for health because media promote standards of beauty that are unrealistic (Grogan, 2016). In particular, media regularly depict incredibly thin girls and women with a body weight and shape unattainable for the average person (Grabe et al., 2008). The ideal has become increasingly thinner over time (Sypeck et al., 2004), with the weight of female models in media often thinner than the criteria for anorexia (Wiseman et al., 1992). Various forms of media support the widespread dissemination of a thin ideal of beauty and extensive exposure to this provides a mechanism to learn the culturally constructed lessons conveyed (Thompson et al., 1999; Groesz et al., 2002). According to cultivation theory, recurrent exposure to media content results in viewers perceiving media portrayals of events or people as reality (Gerbner et al., 2002). This suggests that where there is greater exposure to specific ideals that value “thin” over other body types, an individual may internalize the messages as definitive of societal definitions of attractiveness (O’Brien, 2015). The process of internalization involves the development of positive associations with the idealized model portrayed within the media (Tiggemann, 2002; Thompson et al., 2004). Media portrayals of thin models emphasize perceived reward of unattainable thinness including attractiveness and success (Thompson et al., 1999). The internalized ideal can become problematic because media content often does not mirror reality with many forms of mass media promulgating incomplete, inaccurate and biased content that becomes the standard against which the self and others are judged (Ulaş et al., 2012; Webb et al., 2014; Geraee et al., 2015). Exposure to traditional media, specifically film, television and magazines, results in body dissatisfaction (for a comprehensive review see Grabe et al., 2008) which increases the risk of psychological problems including eating disorders, depression, and low self-esteem (Markey, 2010) and physiological problems such as a weakened immune system (Campisi et al., 2012) and poor sleep quality (Wolniczak et al., 2013).

Although the effect of traditional media on body image disturbance is well-established, media are constantly evolving with new forms such as internet-based, social media increasing in popularity (Qualman, 2009). Social media are a collection of web-based applications that allow the creation and exchange of user-generated content (Kaplan and Haenlein, 2010). Social networking sites (SNSs) are popular social media applications that allow users to create personal profiles of photos, video, audio and blogs which are made available to friends, followers or the public (Kaplan and Haenlein, 2010). Facebook is the largest SNS with two billion users worldwide^1^. Impression and reputation management are important in the use of SNSs because these sites allow users to manage both their social network and social identity (Riva et al., 2016). As a result, physical appearance and self-presentation are central to SNSs (Siibak, 2009), with the most common activities revolving around uploading and looking at photographs (Espinoza and Juvonen, 2011) that are selected based on appearance (Siibak, 2009). Consistent with traditional media, SNS photographs can also be unrealistic portrayals of one’s ideal-self (Meier and Gray, 2014). SNSs center on the use of profile photographs that are malleable, subjective and potentially digitally manipulated (Hancock and Toma, 2009; Toma and Hancock, 2010) similar to the airbrushing and filters used to manipulate traditional media images. Self-presentation online can also be revised by comments and feedback from other users that can alter the view of the self (Moreno and Koff, 2016).

Research has identified an association linking the use of SNSs and body image disturbance (Tiggemann and Slater, 2013). Users of Facebook have higher body dissatisfaction than non-users of the SNS (Stronge et al., 2015). A longitudinal study also found a higher frequency of SNS use predicted increased body dissatisfaction over 1 year later (de Vries et al., 2016). Consistent with findings from traditional media research, viewing idealized SNS profiles results in more negative body image compared to viewing less attractive profile photographs. Furthermore, Smith et al. (2013) explored the effect of maladaptive Facebook use, the tendency to use Facebook for negative social evaluations and/or engage in social comparisons online, on body dissatisfaction and found that maladaptive use predicted increased body dissatisfaction 4 weeks later. Although the relationship linking SNS use and body dissatisfaction is established, the effect of SNSs on body dissatisfaction could be indirect and the mechanisms that explain the association remain unknown (de Vries et al., 2016). The tripartite influence model, which has been applied extensively to traditional media use, proposes that an internalized appearance ideal acts as the mechanism linking socio-cultural influences with body dissatisfaction (Thompson et al., 1999). According to the model, exposure to media causes an individual to internalize cultural ideals of beauty, which lead to appearance dissatisfaction if the individual’s appearance does not match the media portrayal (Thompson et al., 1999; Jones et al., 2004; Shroff and Thompson, 2006). Given the extent of exposure of young people to SNSs it is important to test the generalizability of results from static, traditional media to these new forms (Tiggemann, 2003).

SNSs have the potential to impact the internalization of body image ideals to a greater extent than traditional media because of the characteristics of online information exposure. The real-time and personalized aspect of social media exposure emphasize the potential for social influence to be heightened by comparison to traditional, static and non-personalized exposure that characterize traditional media. Media content on SNSs are available for viewing, creating, editing and, most importantly, sharing, immediately every moment of every day on a plethora of devices (Perloff, 2014). The constant access is paired with an abundance of content, as over 3.2 billion new photographs are uploaded to SNSs every day^2^, which creates exponentially more opportunities for internalization and social comparison than ever achieved via traditional media (Perloff, 2014). Within the context of the tripartite influence model, users of SNSs are exposed online to all three sources of influence (i.e., media, peers, and family) simultaneously in a single medium that may encourage the internalization of body image ideals in a much more profound way than any of three influences in isolation (Slattery, 2013; Meier and Gray, 2014).

Consistent with cultivation theory and the tripartite influence model, the results of studies exploring associations between use of SNSs and internalization of a thin ideal indicate that more frequent exposure to SNSs is associated with higher internalization of a thin ideal (e.g., Tiggemann and Slater, 2013, 2014). Inferences of causation cannot be drawn from correlational data and it is possible that the relationship linking SNS use and thin ideal internalization occurs in reverse, such that women high in thin ideal internalization are drawn to SNSs because they offer a platform to receive instant feedback on appearance. However, the findings of correlational studies assessing SNS use and thin ideal internalization are consistent with experimental research on the effect of traditional media use on body dissatisfaction. Grabe et al. (2008) conducted a meta-analysis of experimental and correlational studies to investigate the relationship between traditional media use and internalization of a thin ideal in women and found a small to moderate effect size (*d* = -0.39) indicating internalization of a thin ideal is associated with women’s use of traditional media. Therefore, in the present study we hypothesized that the use of SNSs will be positively associated with the internalization of a thin ideal. However, researchers have occasionally reported a lack of association with an internalized thin ideal when SNS use was measured as a construct of overall use of a website (Meier and Gray, 2014) whereas studies that operationalized the use of SNSs related specifically to appearance (e.g., posting, commenting, or viewing photographs on SNSs) have found stronger associations with an internalized ideal (McLean et al., 2015). Therefore, how SNS use is operationalized is likely to affect the strength of the relationship.

In the present study we hypothesized that internalization of a thin ideal would be more strongly correlated with exposure to appearance-related content online compared to simple utilization of SNSs. Measuring use of SNS as an overall function of time may be uninformative because of the wide array of content available within websites and applications, some of which may not pertain to the dissemination of cultural ideals of beauty (Meier and Gray, 2014). Given that research into the effect of SNSs on body dissatisfaction is beginning to grow, the results of the emerging literature need to be quantified to understand where the effects lie and the best way to operationalize SNS use to guide future research.

## Method

### Literature Search

This meta-analytic review adheres to the guidelines detailed in the Preferred Reporting Items for Systematic Reviews and Meta-Analyses (PRISMA) statement (Moher et al., 2010). Systematic literature searches were performed in May 2016 in the following electronic databases: PsycINFO, PubMed, Web of Science, Communication and Mass Media Complete, and ProQuest Dissertations and Theses Global. The following key words were searched individually and in various combinations: (social media OR social network OR Web 2.0 OR Facebook) AND (internali#ation OR internali#e OR body image OR appearance AND ideal). Reference lists of included papers were also searched for additional relevant studies.

### Eligibility Criteria

Studies were excluded on the basis of the following criteria: (a) the article did not describe a quantitative methodology; (b) the article did not present original data; (c) the article was not available in full-text; (d) the article was not published between the year 2000 and May 2016, to correspond with the launch of the Web 2.0 platform (Williams et al., 2014); (e) the researchers did not measure nor experimentally manipulate the use of a SNS, with a SNS defined as a web-based service that allows a user to create a profile and interact with a network of other registered users with whom they share a connection (Boyd and Ellison, 2007); (f) the article did not contain a relevant measure of an internalized thin ideal. The data extracted for this meta-analysis were correlations between SNS use and an internalized thin ideal. Consistent with prior meta-analyses (e.g., Grabe et al., 2008), experimental studies were included if both the experimental and control group were exposed to some form of media, and experimental studies that measured internalization at pre-exposure but not post-exposure were also included. Correlational studies were included if the extent of participants’ use of media was assessed, whereas studies that measured media influence (e.g., perceived pressure from SNSs to change appearance or behavior) were excluded because the aim of this meta-analysis was to test the association between use of SNSs and an internalized thin ideal. In one instance both the published manuscript and unpublished thesis of a single study were identified through the searches. The meta-analysis included the published version (Meier and Gray, 2014).

### Study Selection and Data Collection Process

**FIGURE 1:**
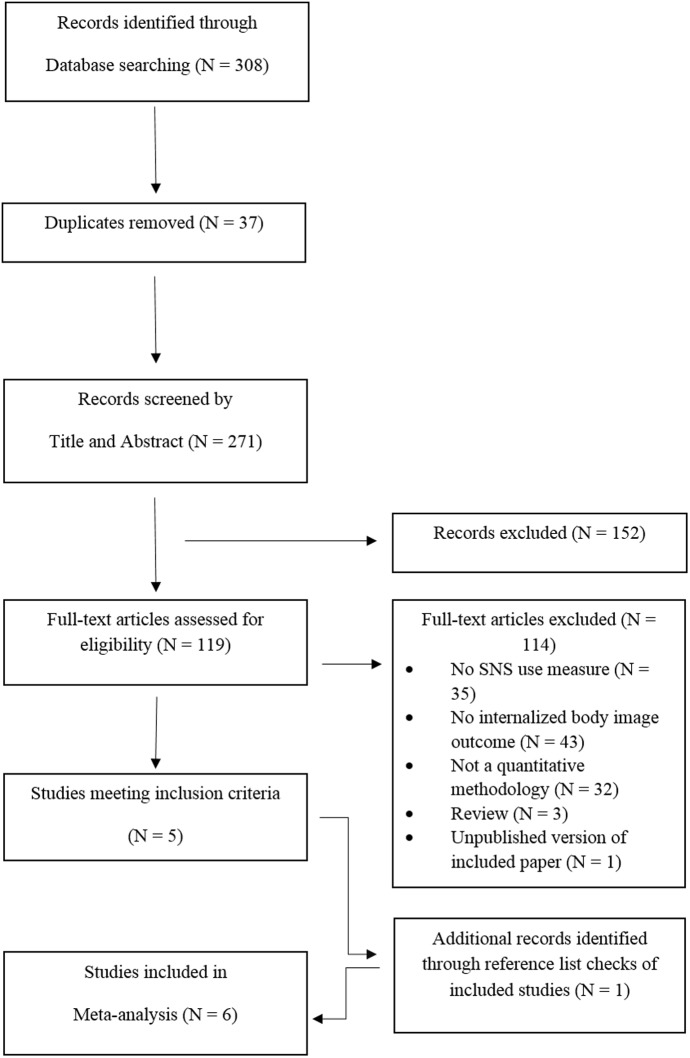
Flow diagram depicting the search protocol and workflow in determining the studies for inclusion in the meta-analysis.

The searches yielded 308 potential articles for review; references were exported to EndNote X7 for screening (see **Figure 1**). After removing 37 duplicates, 271 articles were screened by title and abstract for relevance, which removed a further 152 studies. The full-text of 119 articles were reviewed for compliance with eligibility criteria which removed a further 114 studies. An additional study was included from the reference list of one of the included studies. The first author completed the searches, and an independent researcher (JT) screened a randomly selected subset (10%) of the articles to assess search reliability. One hundred percent agreement was obtained on the included studies.

Data extraction was performed using a standardized form with the following categories: database; author/s; date of publication; journal; country; study design; sample size; gender of participants (%); age of participants (mean, standard deviation); measure of SNS use; internalization outcome measure; and main findings.

### Calculation of Effect Sizes

Effect sizes were calculated using the Hedges and colleagues method (Hedges and Olkin, 1985; Hedges and Vevea, 1998), in which the original correlation coefficients were converted into a standard normal metric using Fisher’s r-to-z transformation (Fisher, 1928). A summary Fisher’s z was then created before being converted back to r and presented as a weighted average of these transformed scores. A positive effect size indicates higher internalization of a thin ideal is associated with more SNS exposure. Correlation values of 0.10, 0.30, and 0.50 represent small, moderate, and large effects, respectively (Cohen, 1988). Random-effects models were used to calculate the average effect-size estimates and confidence intervals (95%) used in this meta-analysis. Random-effects are recommended (Field, 2005) and allow for generalization beyond the studies included in the current meta-analysis (Field and Gillett, 2010) which is preferred given the emerging nature of SNS-related body image research. The analyses were performed using Comprehensive Meta-Analysis Version 3.0 (Borenstein et al., 2014).

Studies included in this meta-analysis contained either a single effect size pertaining to SNS use in general, or contained two effect sizes, one pertaining to the use of a SNS in general and a separate effect size for use of specific appearance-related SNS features. This enabled comparison of the extent to which type of SNS content exposure had a stronger relationship with thin ideal internalization. To calculate the primary outcome for the effect of total use of SNSs, the general SNS use effect was used from each study (see **Table 1**). To explore potential differences between the reported effect sizes for the use of all features on SNSs compared to the reported effect sizes for the use of specific appearance-related features on SNSs, a mean difference comparison effect size was calculated by subtracting the total use of SNSs effect from the appearance-related effect for studies that reported multiple outcomes. A separate meta-analysis was performed on the mean difference scores (Borenstein et al., 2009). Two studies (Tiggemann and Miller, 2010; Tiggemann and Slater, 2013) also included separate effect sizes for the usage of Facebook and MySpace; however, the MySpace effect sizes were excluded from the analyses to maintain consistency in the SNS measured and to ensure generalizability to future research given that MySpace is now generally accepted as a “dead” site (Goodings, 2012, p. 486). The analyses were performed with and without these effect sizes and the statistical significance was not affected.

**Table 1:** Descriptive characteristics of the studies included in the analyses.

Study	Type of SNS use	*N*	*r*	*M* age (years)	Study design	Country	Measurement of SNS use	Internalization measure
Cohen and Blaszczynski, 2015	Overall use	193	0.12	19.32	Experimental	AUS	Time per day	Sociocultural Attitudes Toward Appearance Questionnaire–3: Pressure subscale (Thompson et al., 2004)
	Appearance-related features		0.17					
McLean et al., 2015	Overall use	101	0.16	13.13	Correlational	AUS	Engagement with SNSs	Sociocultural Attitudes Toward Appearance Questionnaire-4: Internalization: Thin/Low Body Fat subscale (Schaefer et al., 2015)
	Appearance-related features		0.24					
Meier and Gray, 2014	Overall use	103	0.12	15.40	Correlational	USA	Time per day using defined categories	Sociocultural Internalization of Appearance Questionnaire for Adolescents (Keery et al., 2004)
	Appearance-related features	89^a^	0.36					
Tiggemann and Miller, 2010	Overall use	156	0.18	14.90	Correlational	AUS	Time per day	Sociocultural Internalization of Appearance Questionnaire for Adolescents (Keery et al., 2004)
	Appearance-related features		0.30					
Tiggemann and Slater, 2014	Overall use	189	0.32	11.50	Correlational	AUS	Time per day	Sociocultural Internalization of Media Ideals Scale (Jones et al., 2004)
Tiggemann and Slater, 2013	Overall use	1087	0.16	13.70	Correlational	AUS	Time per day	Three items from the Sociocultural Attitudes Toward Appearance Questionnaire (Thompson et al., 2004)

*SNS, social networking site; *N*, total sample; *r*, effect size; AUS, Australia; USA, United States*.

^a^*Meier and Gray (2014) appearance-related features N has 89 participants because the measure of appearance-related features includes only the participants who reported as photo-sharers in a previous measure*.

### Publication Bias

To examine potential publication bias, Rosenthal’s and Orwin’s Fail-safe N (Orwin, 1983; Rosenthal, 1991) were calculated to determine the number of un-retrieved studies with an effect size of zero that were needed to make the cumulative effect non-significant (*p* > 0.05) or trivial (*r* < 0.01). Egger’s regression coefficient (Egger et al., 1997) was calculated to asses for small study bias and Duval and Tweedie’s trim and-fill method (Duval and Tweedie, 2000) with a random effects model was applied to estimate the number of studies missing due to funnel plot asymmetry.

## Results

### Descriptive Results

Six studies based on 1,829 participants were included in the meta-analysis (see **Table 1**). Year of publication ranged from 2010 to 2015. Five (83.3%) of the studies were conducted in Australia and one was from the United States of America. Study sample sizes ranged from 101 to 1,087 participants and consisted of females ranging in age from 10 to 46 years. Most studies were correlational in design and the mean age of participants sampled in the studies ranged from 11.5 to 19.32 years. Most studies recruited secondary school females (66.6%), with one study recruiting primary school girls and one study recruiting university students. Of the six included studies, all reported a measure of overall use of a SNS, and four studies reported measures of both overall use of a SNS and use of appearance-related features on a SNS.

### Measurement of SNS Use

Social media use was defined consistently in most studies as the use of a specific SNS; Facebook. One study specifically measured multiple social media platforms to account for changing trends in SNS use and to ensure generalizability to future research (McLean et al., 2015). Researchers specifically developed items for the measurement of total SNS use in each of the included studies. Items used to measure overall use of a SNS typically asked participants to report the average duration of time spent on a SNS over a specified period in minutes or hours. One study asked participants to select their time spent using Facebook from pre-determined categories based on the national averages for adolescent SNS use (Meier and Gray, 2014). One study also measured overall engagement with SNSs as the number of forms of social media that a participant used (McLean et al., 2015). The effect size of this study was consistent with the studies that asked participants to indicate their time spent using SNSs in minutes or hours (see **Table 1**).

### Measurement of Appearance-Related Features on SNSs

Researchers also developed items measuring use of specific appearance-related features on a SNS for the purposes of the studies. Measurement of appearance-related features on a SNS typically asked participants to report how often they engaged in specific activities related to appearance (e.g., viewing friends’ photos). In one study, the researchers developed and validated a questionnaire (The Facebook Questionnaire) to measure Facebook usage generally and Facebook appearance-related feature use specifically (Meier and Gray, 2014). Appearance measures assessed factors such as number of self-photos (“selfies”) a user posted to a SNS and how long a user spent viewing profiles or comments from other users. Another developed the Social Media and Digital Communications Scale that assessed broad uses of all SNSs rather than use of one specific site (McLean et al., 2015). One study measured an Internet appearance exposure score which included the use of the most popular websites used by participants, many of which were SNSs or involved an element of social networking (Tiggemann and Miller, 2010). Although this effect size did not exclusively reflect use of SNSs, the analyses were performed with and without this effect size and the statistical significance was not affected; therefore the effect size was retained in the data analyses.

### Measurement of Thin Ideal Internalization

Standardized measures were used to assess the thin ideal internalization in all of the included studies (see **Table 1**). Three studies measured internalization of a thin ideal with a subscale of the Sociocultural Attitudes Toward Appearance Questionnaire (Tiggemann and Slater, 2013; Cohen and Blaszczynski, 2015; McLean et al., 2015). A further study utilized the Sociocultural Internalization of Media Ideals Scale which was adapted from the Sociocultural Attitudes Toward Appearance Questionnaire (Jones et al., 2004). Two studies measured internalization with a measure specifically designed for use with adolescents; the Sociocultural Internalization of Appearance Questionnaire for Adolescents (Tiggemann and Miller, 2010; Meier and Gray, 2014).

### Effect Size Analyses

#### Total Use of SNSs

**FIGURE 2:**
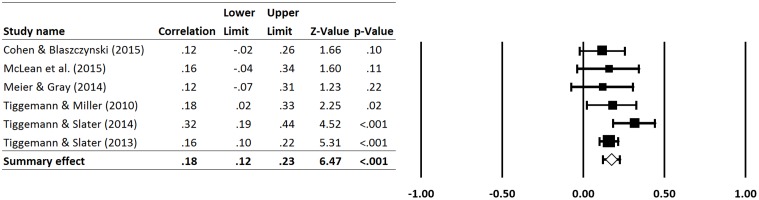
Summary effect sizes of the relationship between total use of SNSs and the internalization of a thin ideal.

All six of the effect sizes were positive. As can be seen in **Figure 2**, the weighted mean effect size for the relationship between overall SNS use and internalization of a thin ideal, averaged over 6 independent effect sizes, was 0.18 (95% CI 0.12 to 0.23, *p* < 0.001), representing a small to moderate effect by Cohen’s criteria (Cohen, 1988). The positive effect indicated that more use of SNSs was associated with significantly higher internalization of a thin ideal in females. The proportion of the observed variance that reflects real differences between studies was 11.13% indicating a small level of non-significant heterogeneity (*p* = 0.344).

The analyses were also performed by calculating a mean effect size for each study that contained a separate effect size for both total use of a SNS and an appearance-related SNS use. The weighted mean effect size increased significantly but marginally (*r* = 0.21; 95% CI 0.15 to 0.28, *p* < 0.001); however, all six of the effect sizes were statistically significant when a combined mean for each study with multiple outcomes was calculated.

#### Comparing Study Outcomes

Four studies reported an effect size for total use of a SNS as well as separate effect for the use of specific appearance-related content on a SNS (see **Table 1**). The statistically significant difference in the size of effect between appearance specific use of SNSs and general use of SNSs demonstrated a larger effect for the former. The use of appearance-related content resulted in a 0.11 (95% CI 0.03 to 0.19) increase in the mean effect size compared to the total use of a SNS (*p* = 0.009). The positive effect indicated that females reported significantly greater internalization of a thin ideal when SNS use was measured as a function of specific appearance-related features rather than for the use of all features on SNSs.

#### Publication Bias

Rosenthal’s and Orwin’s Fail-safe N calculations identified that between 111 and 153 un-retrieved studies with an effect size of zero were needed to conclude that the cumulative effect observed was non-significant or trivial. Egger’s test for publication bias was non-significant (*p* = 0.159), indicating no evidence of asymmetry. Duval and Tweedie’s trim and-fill method was also applied, and no studies were found to be missing and the point estimate and 95% confidence interval remained unchanged. Collectively, these analyses suggest that it is unlikely that the results of this meta-analysis are due to publication bias.

## Discussion

The current meta-analysis measured the relationship between using SNSs and the internalization of a thin ideal among females across six studies. The results revealed using SNSs positively correlated with internalization. The relationship between use of SNSs and perceptions of body attractiveness raises concern for the well-being of its users given the photographs promulgated can be digitally altered to adhere to unrealistic beauty ideals (Mabe et al., 2014) and the fact that over one billion people are exposed to content on Facebook daily^3^. The size of the problem is highlighted by the 3.2 billion new photographs uploaded every day on SNSs^4^.

The larger size of the correlation between exposure to appearance-related content versus general exposure to all content is consistent with appearance being influenced by what is seen rather than just read on SNSs. Consistent with cultivation theory, recurrent exposure to photographs and messages on SNSs appears to result in the adoption of these images as attractive. This suggests that a user’s interaction with these appearance-related features is of the greatest importance to body image research. The greater effect size found when measuring appearance-related features on SNSs has implications for future measurement of SNS use which should target the appearance-related content rather than the overall use of a SNS. Moreover, more fine-grained analysis of the use of specific appearance related content and how it impacts on body-image internalization may highlight content at most risk of creating distorted views.

The finding of a small to moderate effect size is consistent with a prior meta-analysis of the association between traditional media exposure and internalization of a thin ideal in women (Grabe et al., 2008). According to the tripartite influence model, exposure to traditional media results in internalization of a thin ideal which in turn predicts body dissatisfaction. The findings of this meta-analysis suggest that the tripartite influence model may be applicable to new media exposure, suggesting that internalization of a thin ideal could be a mechanism in the relationship between SNS use and body dissatisfaction. The use of SNSs could be an additional socio-cultural predictor of body dissatisfaction that is a concern for the development of eating disorder symptomology given the far easier accessibility of social media. Although we did not explore the association between a thin ideal internalized from SNS use and body dissatisfaction in this meta-analysis, it is plausible that the overall effect on body dissatisfaction will be evident given the findings of this study are consistent with the previous two decades of body image research that support the model in traditional media (Karazsia et al., 2013). Research exploring SNS use and body dissatisfaction also supports this conclusion by identifying that more SNS use is associated with higher body dissatisfaction across males and females (e.g., Stronge et al., 2015; de Vries et al., 2016).

Given the knowledge that social media use is associated with body image, the results of this study can also be used to inform media literacy interventions that commonly focus upon traditional media. These interventions can be expanded to educate adolescents about the way social media portrays body image ideals and the often unrealistic nature of the portrayals. The photographs and messages on social media are often perceived as realistic because they are traditionally being disseminated from within a network of one’s peers rather than from groups or individuals outside the network. Many people have become educated in the way advertising images and celebrity images are manipulated; however, this knowledge may not translate into social media images because of the assumption that the images are of one’s peers who have shared resources and lifestyles (Perloff, 2014). Media literacy relating to SNS can educate users about the way photographs on SNSs can be manipulated through various applications and techniques to quickly and easily alter photographs and portray idealized creations of the self. For example, image enhancement features such as photographic filters are often built directly into the camera and photography software on mobile phones, tablets, and computers while countless applications are also available to further enhance personal photographs to a professional level prior to their uploading to social media. Furthermore, SNSs could be used as a platform to create and disseminate positive body image messages and interventions.

Future research should interpret the results of this meta-analysis in relation to the following limitations. The emerging nature of SNS research restricted the meta-analysis to a small number of studies that reduced the statistical power to determine the heterogeneity and precision of the effect sizes; however, no evidence of publication bias was found. The included studies were predominately correlational, and although causation cannot be inferred from correlational data, it has been demonstrated in experimental research that exposure to thin ideal images in traditional media results in poorer body image outcomes than exposure to average weight models, plus-size models, or neutral objects (Groesz et al., 2002). There was some minor variability in the measurement of SNS use that was likely due to the authors creating the items used in each study. Most of the included studies focused on Facebook as the SNS of interest. Although this approach is warranted given Facebook is the largest and most popular SNS, it will be important for future research to assess the use of multiple SNSs. The findings of this meta-analysis indicated that exposure to appearance-related content on SNSs is of the greatest concern for body image disturbance and future research should aim to address the use of primarily photograph-based platforms including Instagram and Snapchat. The included studies primarily focused on Facebook which allowed for consistency among the measurement of appearance-related feature use; however, as future research begins to explore the use of multiple SNSs it will be important to refine the measurement for appearance-based features to allow these measures to become more generalizable because features may differ among platforms.

Future research should aim to broaden the samples used in SNS body image research beyond female participants; this could allow for the exploration of additional appearance ideals (e.g., mesomorphic ideal; tanned ideal). The included studies exclusively sampled female participants despite body dissatisfaction among males being associated with SNS use to the same extent as females’ (de Vries et al., 2016). The association with male body dissatisfaction warrants further exploration, particularly on SNSs where fitness has become a common theme as over 70% of profiles reference fitness behaviors and Facebook itself tailors the advertisements its users see by pairing key words and phrases from users’ profiles with fitness advertisements (Villiard and Moreno, 2012).

Further research is also necessary to test potential differences in the relationship for girls and women of different ages. Previous research has found thin-ideal images affect body image in girls under 19 years of age to a greater extent than older participants (Groesz et al., 2002). Although the current study did not include enough samples with primary school aged participants to effectively draw comparisons, the reported relationship was highest in the sample of 10 to 12 year old girls. The finding is concerning given that the primary school aged girls were younger than the minimum age to register a SNS account^5^.

In summary, this review establishes that use of SNSs is related to female body image internalization. Exposure to appearance-related content on SNSs had a greater relationship with the internalization of a thin ideal than overall use of the broad array of features on SNSs which indicated the appearance-related features should be targeted in future interventions to help reduce the risk of body dissatisfaction.

## Author Contributions

All persons who meet authorship criteria are listed as authors, and all authors certify that they have participated sufficiently in the work to take public responsibility for the content, including participation in the concept, design, analysis, writing, or revision of the manuscript.

## Conflict of Interest Statement

The authors declare that the research was conducted in the absence of any commercial or financial relationships that could be construed as a potential conflict of interest.

## Footnotes

1 “Two Billion People Coming Together on Facebook,” Mike Nowak and Guillermo Spiller, accessed July 05, 2017, https://newsroom.fb.com/news/2017/06/two-billion-people-coming-together-on-facebook/.

2 “12 global trends every marketer needs to know now,” Cameron Brain, last modified September 10, 2016, https://www.linkedin.com/pulse/12-global-trends-every-marketer-needs-know-now-cameron-brain.

3 “Company Info,” Facebook, accessed July 5, 2017, http://newsroom.fb.com/company-info/

4 “12 global trends every marketer needs to know now,” Cameron Brain, last modified September 10, 2016, https://www.linkedin.com/pulse/12-global-trends-every-marketer-needs-know-now-cameron-brain

5 “How do I report a child under the age of 14 in South Korea or Spain?”, Facebook, accessed October 26, 2016, https://www.facebook.com/help/100532533374396?sr=4&query=minimum+age&sid=0qp4Muc3C0gYWqsCr.
